# Experience in Post-Bariatric Abdominoplasty for Patients with Significant Weight Loss: A Prospective Study

**DOI:** 10.3390/jpm14070681

**Published:** 2024-06-25

**Authors:** Roberto Cuomo, Claudia Cuccaro, Ishith Seth, Warren M. Rozen, Maria Giovanna Vastarella, Giuseppe A. G. Lombardo, Francesco Ciancio, Domenico Pagliara, Gorizio Pieretti, Feliciano Ciccarelli

**Affiliations:** 1 Plastic Surgery Unit, Department of Medicine, Surgery and Neuroscience, University of Siena, 53100 Siena, Italy; 2 Organization of Hospital Services Unit, Santa Maria Alle Scotte Hospital, 53100 Siena, Italy; 3 Department of Plastic Surgery, Peninsula Health, Melbourne, VIC 3199, Australia; 4 Faculty of Science, Medicine, and Health, Central Clinical School at Monash University, The Alfred Centre, 99 Commercial Rd, Melbourne, VIC 3004, Australia; 5 Department of Woman, Child and General and Specialized Surgery, University of Campania “Luigi Vanvitelli”, 80138 Naples, Italy; 6 Cannizzaro Hospital of Catania, Kore University of Enna, 94100 Catania, Italy; 7 Cannizzaro Hospital of Catania, 95123 Catania, Italy; 8 Gynecology and Breast Care Center, Mater Olbia Hospital, 07026 Olbia, Italy; 9 Plastic and Reconstructive Surgery Unit, Multidisciplinary, Department of Medical-Surgical and Dental Specialties, University of Campania Luigi Vanvitelli, 80138 Naples, Italy; 10 Department of Plastic Surgery, Villa dei Fiori Hospital, 80011 Naples, Italy

**Keywords:** abdominoplasty, tummy tuck, post bariatric, plastic surgery, body contouring

## Abstract

Background: Abdominoplasty is a critical aesthetic and functional procedure for individuals who have undergone massive weight loss. Numerous techniques have been proposed to optimize aesthetic results while minimizing complications. Methods: This prospective study examined 500 patients who underwent abdominoplasty during body-contouring procedures between 1 January 2018 and 31 December 2021 at a tertiary center. The Skin–Adipose Tissue–Muscle (SAM) protocol was employed to analyze the operative strategies and complication rates and compare them with the existing literature. Furthermore, patient satisfaction and aesthetic outcomes were measured one year post-operation using a comprehensive four-point questionnaire evaluated by the patients themselves and two independent surgeons. Results: Participants had an average age of 34.8 years and a mean BMI of 31.1 kg/m^2^. The surgeries included 328 full abdominoplasties and 172 T-inverted abdominoplasties. Notable complications included wound infection (4%), wound dehiscence (8.6%), tissue necrosis (0.6%), seroma (8.4%), and hematoma (2.6%). A higher BMI was correlated with an increased risk of complications and lower patient satisfaction. Data analysis was performed using Stata version 18 software. Conclusions: The increasing prevalence of obesity highlights an urgent need for more bariatric surgeries and subsequent abdominoplasties to mitigate the effects of massive weight loss. A crucial link between elevated BMI and a heightened risk of postoperative complications, emphasizing the necessity for standardized surgical protocols tailored to individuals with higher BMI, was noted. Innovatively, future studies must further investigate the intricate dynamics between BMI and surgical risks. Exploring and establishing uniform, adaptive surgical guidelines promise to revolutionize patient care by significantly reducing complications and enhancing recovery and satisfaction following abdominoplasty.

## 1. Introduction

As obesity rates soar globally, bariatric surgeries have emerged as a pivotal measure for achieving substantial weight loss in individuals with morbid obesity. These procedures are instrumental in weight management and alleviating related health issues. However, they often lead to excessive skin and soft tissue redundancy post-operatively, necessitating subsequent body contouring surgeries to address the physical transformations. The increasing need for such surgeries underscores the multifaceted impact of massive weight loss, encompassing aesthetic, functional, and psychosocial dimensions. Specifically, abdominoplasty is increasingly sought after by those who have undergone dramatic weight reduction following bariatric surgery, offering a surgical remedy to these post-operative challenges [1,2].

Significant weight reduction typically leads to extensive skin laxity in multiple body areas and often includes the separation of rectus muscles, known as rectus muscle diastasis. To remedy these aesthetic and functional concerns, patients frequently seek surgical solutions. Among these, abdominal dermolipectomy is a prominent choice for its comprehensive approach to removing excess tissue and restoring the body’s contour [3,4,5,6,7]. Different surgical techniques have been proposed over time, using horizontal and/or vertical incisions, sometimes combined with liposuction, and the recent focus on using new state-of-the-art technologies [8,9,10,11].

The primary aim of this study was to evaluate the efficacy and safety of various abdominoplasty techniques in patients who have undergone massive weight loss, particularly following bariatric surgery. By implementing the Skin–Adipose Tissue–Muscle (SAM) protocol across a tertiary center, we sought to identify the optimal surgical strategies that minimize postoperative complications while maximizing aesthetic and functional outcomes. A secondary aim was to analyze the correlation between body mass index (BMI) and the incidence of complications, patient satisfaction, and aesthetic results. Furthermore, the study aimed to assess patient satisfaction and aesthetic outcomes one year post-operation using a comprehensive four-point questionnaire evaluated by the patients and two independent surgeons. Secondary outcomes included the evaluation of specific complication rates, such as wound infection, wound dehiscence, tissue necrosis, seroma, and hematoma, and the effectiveness of advanced surgical techniques, including 3D imaging for surgical planning and state-of-the-art wound closure methods. By addressing these aims, the study contributes to the evolving field of post-bariatric body contouring, providing valuable insights into tailored surgical protocols and improving patient care outcomes.

## 2. Materials and Methods

This study received ethical clearance from the Ethical Committee of Villa Dei Fiori Accredited Hospital (HREC of Casa Di Cura Villa Dei Fiori Approval Code: PROPT M.2019_11_T), aligning with the rigorous principles of the Declaration of Helsinki. Each participant granted informed consent, thereby authorizing the surgical procedures, their participation in the research, and the subsequent dissemination of their anonymized results and photographs. Our prospective analysis focused on individuals who received abdominoplasty post-bariatric surgery at our tertiary hospital from 1 January 2018 to 31 December 2021. We carefully evaluated each patient’s cardiovascular and pulmonary health, including conditions like coronary heart disease and chronic obstructive pulmonary disease, to gain a holistic view of their medical history. To ensure a concentrated study scope, we excluded patients with abdominal hernia defects. The abdominoplasties were conducted in strict accordance with our institutional SAM protocol, a specialized surgical guideline that accounts for individual variations in skin laxity, adipose tissue volume, and muscle integrity. This precise and personalized approach was designed to provide superior care tailored to each patient’s unique anatomical needs. We gauged patient satisfaction one year after the surgery through a comprehensive four-point scale questionnaire aimed at capturing detailed insights into their surgical journey. Furthermore, two experienced surgeons independently assessed the surgical outcomes, focusing on skin and fat correction, overall abdominal aesthetics, and umbilicus placement utilizing the same four-point scale. Beyond measuring patient satisfaction and aesthetic success, this study conducted a rigorous comparison of complication rates against the existing literature, placing our findings within a wider surgical safety and efficacy context. Statistical analysis was performed using Stata version 18 software.

### 2.1. Inclusion Criteria

Patients previously submitted to bariatric surgery, with a Pittsburgh score upper 10, and a stable weight for at least 6 months were included in plastic surgery procedures.

### 2.2. Operative Technique

**Figure 1:**
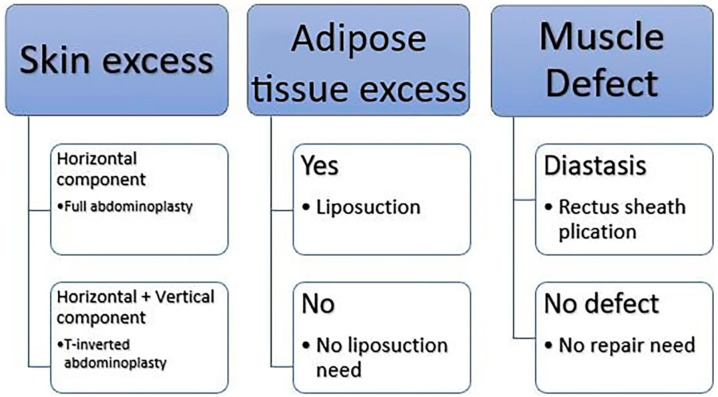
An overview of the surgical decision-making process.

The surgical decision-making process was based on three different anatomical features: skin laxity, adipose tissue excess, and muscle continence (Figure 1). This triad of considerations allowed for a nuanced and tailored strategy for each patient. Skin laxity was evaluated to understand the amount of sagging and excess skin, which varies significantly among individuals and impacts the extent of tissue removal required. Adipose tissue excess was assessed to determine the volume and distribution of fat deposits, guiding the need for an approach to liposuction or direct excision. Lastly, muscle continence was considered crucial for restoring the abdominal wall’s functionality and aesthetics, especially in addressing diastasis recti, which is common in post-bariatric patients.

### 2.3. Skin Laxity

**Figure 2:**
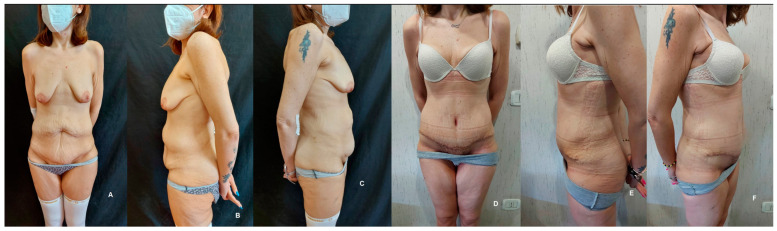
Comparative preoperative (**A**–**C**) and postoperative (**D**–**F**) frontal and lateral views of a patient undergoing abdominoplasty and umbilical transposition. This case highlights the reduction in excessive abdominal skin in the hypogastrium area, categorized under a Pittsburgh scale of abdomen, flanks, and mons pubis < 7.

**Figure 3:**
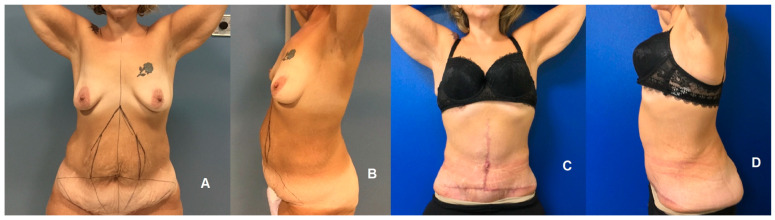
Comparative preoperative (**A**,**B**) and postoperative (**C**,**D**) frontal and lateral views of a patient undergoing complete T-Inverted abdominoplasty. This procedure, characterized by a combination of horizontal and vertical scars, is indicated for patients with a Pittsburgh scale of abdomen, flanks, and mons pubis > 7.

Skin excess was assessed using the Pittsburgh scale [12]. For significant ptosis or abdominal skin excess at the epigastric and hypogastric regions (Pittsburgh scale of abdomen, flanks, and mons pubis < 7), a comprehensive tummy tuck including umbilical transposition was employed (Figure 2). In instances of pronounced vertical skin surplus (Pittsburgh scale of abdomen, flanks, and mons pubis > 7), a full T-inverted abdominoplasty was conducted, utilizing both horizontal and vertical incisions for optimal results (Figure 3). This stratified approach ensured tailored interventions aligned with the severity and nature of skin laxity.

### 2.4. Adipose Tissue Excess

**Figure 4:**
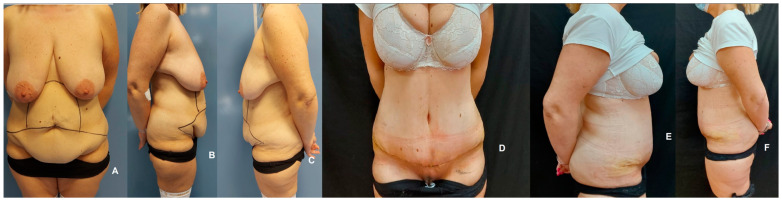
Comparative preoperative (**A**–**C**) and postoperative (**D**–**F**) lateral and frontal views of a patient undergoing tummy tuck and liposuction. This case demonstrates the reduction in skin tissue in the flank region, illustrating the procedure’s effectiveness.

Patients exhibiting notable fat accumulation, especially in the epigastric region, undergo liposuction as an initial step (Figure 4). Pre-operatively, liposuction areas are marked while the patient stands, ensuring precise targeting during surgery. Incisions for cannula insertion are strategically made near the umbilicus for the mesogastric and epigastric areas and on either side of the inguinal region for flank liposuction. This positioning is crucial as it aligns with the planned skin excision zones. Prior to liposuction, these targeted areas are infiltrated with a solution containing 1 mg of epinephrine in 1000 mL of saline, allowing 20 min for it to take effect. Subsequently, comprehensive liposuction is performed on both deep and superficial layers using 4/5 mm blunt-tip cannulas. Importantly, this liposuction process excludes areas designated for skin resection, ensuring a harmonious and effective reduction in excess fat while preserving the integrity of the areas set for skin removal. This approach optimizes the contouring process, enhancing the overall aesthetic outcome of the abdominoplasty.

### 2.5. Muscle Continence

**Figure 5:**
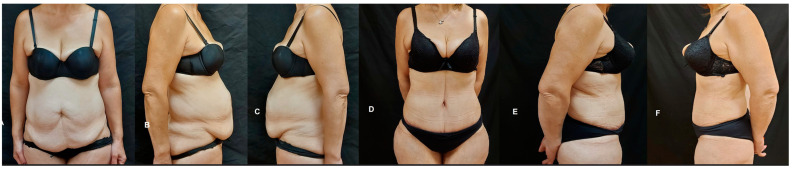
Comparative preoperative (**A**–**C**) and postoperative (**D**–**F**) lateral and frontal views of a patient with rectus diastasis. This case depicts the importance of plication using resorbable or non-resorbable sutures to correct anterior bulging and achieve a slender body profile.

A comprehensive preoperative evaluation, including clinical examination and ultrasonography of the rectus abdominis, is conducted to detect any diastasis. When diastasis is identified, we execute rectus sheath plication extending from the xiphoid process to the pubic symphysis. This procedure involves a continuous double-stranded 0-Vycril (Polyglactin 910) suture. Plicating the diastasis is vital for rectifying anterior abdominal protrusion and sculpting a more streamlined body silhouette (Figure 5). This method ensures that both the aesthetic and functional aspects of muscle continence are effectively addressed, enhancing the overall outcome of the abdominoplasty.

### 2.6. Surgical Technique

Preoperative Marking: While the patient stood with arms resting alongside the body, bilateral markings were made approximately 3 cm below and 3 cm to the side of the anterosuperior iliac spine. An anticipated final scar line—straight, curved, or stepped—was marked suprapubically, connecting the two points. From here, two curved lines extended to the umbilicus, demarcating an oval region. If a great amount of skin was present, a vertical incision (xypho-pubical) was also planned to remove a median island of skin.

Operative Preparations: Patients were equipped with sequential compression devices on the lower extremities. Prophylactic antibiotics were administered upon the insertion of a Foley catheter. Notably, anticoagulation medications were abstained from. All surgical procedures were executed under spinal anesthesia. 

Incisions and Dissections: The procedure commenced with the umbilicus being circumscribed and released. Incisions on both sides of the marked oval area were deepened to the muscular fascia level. Preferring the lateral side for its thinner skin, which simplifies the identification of the muscular fascia, the proximal flap was undermined. Electrosurgical pre-aponeurotic deep dissection was carried out in a 60 W coagulation mode. 

Suturing and Flap Management: A continuous double-stranded 0-Vycril (Ethicon) suture was employed for rectus sheath plication. The umbilicus was then repositioned and affixed to the fascial plane. To optimize flap advancement, the patient’s back was raised to a 40 degree angle, the anterior abdominal flap was split along the center, and superfluous dermal fat flaps were assessed and removed. The pubic flap was subsequently debulked to equate the flap thicknesses. 

Flap Fixation and Drainage: The abdominal and pubic flaps were anchored to the pubic periosteum, ensuring deep and non-palpable knots to deter the scar from ascending. Dual closed suction drains were positioned via distinct incisions in the pubic region, extending to the lateral thigh, aiding fluid output observation and lessening post-operative discomfort. The abdominal flap was then medialised, mitigating dog ears at the incision’s outer corners and curtailing the scar’s width. 

Closure: The skin was closed in layers: initially, the superficial fascia system was sutured with 2-0 Vycril (Ethicon), followed by suturing of the deep dermis using 2-0 Monocryl (Ethicon) and finalization with an intracuticular 3-0 Monocryl (Ethicon) suture. 

Postoperative Care: A compressive dressing was applied for six days, succeeded by a compressive body scabbard for a month. This minimizes seroma risk by decreasing the detached tissue’s dead space, promoting tissue–fascia adhesion. Patients were advised complete bed rest on the first post-operative day, with the addition of an antithrombotic device or prophylactic enoxaparin sodium, contingent on the Caprini venous thromboembolism risk assessment. Drains were removed once fluid output decreased below 50 cc over 24 h. The patients were followed up for a mean period of 1 year, during which the complication rate and patient satisfaction were evaluated and recorded. All the operated patients were interviewed to assess the level of satisfaction in the long term concerning the correction achieved and the final scar. Pre- and post-operative abdomen assessment was photographically documented.

### 2.7. Outcomes Assessment

One year post-surgery, patients completed a four-point questionnaire to assess their satisfaction. Additionally, two independent surgeons evaluated the surgical outcomes based on three criteria: correction of skin/fat excess (encompassing the size and shape of the abdomen), the overall appearance of the abdomen, and the positioning of the umbilicus. A native speaker translated these questionnaires before patient submission (see Appendix A).

## 3. Results

During the study, 500 patients were enrolled, comprising 382 females and 118 males. The average age was 34.8 years, ranging from 25 to 61. The mean BMI was 31.1, spanning from 25 to 48, with an average weight loss of 60.23 kg, ranging from 30 to 89.5 kg; 22% (110 patients) were active smokers (Table 1). In this cohort, 5.2% (26 patients) had type 2 diabetes, 2.8% (14 patients) experienced cardiovascular diseases, 11.4% (57 patients) had hypertension, and 4.4% (22 patients) suffered from pulmonary conditions like asthma or COPD. Each participant had previously undergone bariatric surgery, detailed as 203 laparoscopic adjustable gastric bands, 80 gastric sleeves, 194 gastric bypasses, and 23 with a combination of gastric bypasses and sleeves. The procedures included 128 T-inverted abdominoplasties and 372 horizontal abdominoplasties. 

Table 2 delineates postoperative complication rates between traditional and inverted-T abdominoplasty procedures. Traditional abdominoplasty was performed on 372 patients (74%), whereas the inverted-T procedure was applied to 128 patients (26%). Notable complications included wound infection (1.8% vs. 2.2%), wound dehiscence (4% vs. 4.6%), seroma (3.6% vs. 4.8%), hematoma (1% vs. 1.6%), tissue necrosis (0% vs. 0.6%), and deep venous thrombosis (0.2% vs. 0%). The mean BMI for patients experiencing complications showed a significant increase, with a *p*-value indicating statistical significance in most categories except for tissue necrosis and deep venous thrombosis. The multinomial logistic regression analysis (Table 3) further emphasizes the significant correlation between BMI and postoperative complications. For instance, the regression coefficients for BMI in relation to seroma, hematoma, infection, and wound dehiscence were highly significant (*p* < 0.001), indicating that higher BMI significantly elevates the likelihood of these complications. Conversely, tissue necrosis did not show a significant association with BMI (*p* = 0.445), suggesting other factors may be more influential in this complication. Overall, the data underscore that higher BMI is a robust predictor of increased postoperative complications in abdominoplasty, necessitating tailored surgical strategies and meticulous patient management to mitigate these risks.

### 3.1. Clinical Outcome and Complication Rates after Abdominoplasty

No fatalities were reported among the 500 patients. Complications included wound infections in 4% (20 patients), wound dehiscence in 8.6% (43 patients), tissue necrosis in 0.6% (3 patients), seromas in 8.4% (42 patients), and hematomas in 2.6% (13 patients). One case of deep venous thrombosis was recorded. The total complication rate was 15% (75 patients). Of the infections, 18 presented as erythema and two as erythema with secretion, all resolving within 10 days following targeted antibiotic therapy based on swab cultures. Wound dehiscence, predominantly located on the distal and median parts of the incision, was treated with traditional medications or negative pressure wound therapy (NPWT) based on size, and two cases required surgical revision. All seromas were managed in an outpatient setting. No reoperations were needed. Four patients underwent surgical scar revision under local anesthesia. This detailed evaluation of clinical outcomes and complication rates provides a comprehensive view of the abdominoplasty post-operative scenario.

### 3.2. Aesthetic Outcome Evaluation

One year post-surgery, patients and surgeons completed a point questionnaire to assess their satisfaction. Additionally, two independent surgeons evaluated the surgical outcomes based on three criteria: correction of skin/fat excess (encompassing the size and shape of the abdomen), the overall appearance of the abdomen, and the positioning of the umbilicus. The average scores reported were 12.453 ± 0.497 from patients and 11.895 ± 0.201 from surgeons. 

### 3.3. Statistical Analysis

The Wilcoxon test analyzed various factors such as age, BMI, patient and surgeon scores, and weight reduction, comparing them with the type of abdominoplasty, smoking status, complication occurrence, and comorbidity presence. A notable correlation emerged between higher BMIs and an increased incidence of complications. Additionally, a significant link was found between higher BMIs and the presence of comorbidities within the study group. The Kruskal–Wallis test, ideal for comparing multiple groups’ means, identified significant disparities between patient age, BMI, and weight reduction when cross-referenced with types of comorbidity and complication. This analysis confirmed significant variances between age and comorbidity, as well as between BMI, comorbidity, and complication (*p* = 0.006). Further exploration using the Wilcoxon test affirmed that higher BMIs correlate with an elevated risk of severe complications such as tissue necrosis and wound dehiscence. 

Spearman and Pearson tests linked age and BMI, revealing that higher BMIs tend to associate with older ages, though this correlation was relatively weak (RHO = 0.12 and 0.13, respectively). In contrast, a Spearman test between BMI with comorbidities (Supplementary Figure S1) and complications showed a strong association (RHO = 0.65), indicating that an increase in BMI significantly correlates with both a higher number of comorbidities and complications. Comparative analysis of long-term satisfaction scores from patients and surgeons unveiled that higher BMIs generally lead to lower satisfaction scores from both parties, suggesting that the effectiveness of these procedures in body reshaping diminishes with higher BMI levels (refer to Supplementary Figure S1 and Supplementary Figure S2 for detailed results). This nuanced statistical examination provides a deeper understanding of the multifaceted impacts of BMI on post-abdominoplasty outcomes.

## 4. Discussion

In this study of 500 patients who underwent post-bariatric abdominoplasty, several key observations were made. A significant finding was the confirmation of a direct correlation between higher BMI levels and an increased risk of postoperative complications. This link is further substantiated by the observed association between higher BMIs and a higher incidence of comorbidities, including type 2 diabetes and cardiovascular diseases, aligning with broader medical findings [9]. Notably, the variance in satisfaction scores from both surgeons and patients for those with higher BMIs highlighted the complexities and challenges in achieving desired aesthetic and functional results in this group. This study also unearthed a mild yet intriguing correlation between increased age and higher BMI, suggesting a potential interaction between age-related metabolic changes and weight management. These insights emphasize the need for tailored surgical approaches and enhanced preoperative preparations for patients with higher BMIs. Investigating the relationship between age and BMI in greater depth could provide valuable insights into effective patient care and optimal timing for surgical interventions. Future research focusing on this age–BMI relationship could unveil new strategies for comprehensive patient management, thereby improving overall outcomes in post-bariatric abdominoplasty.

The growing trend in body contouring surgeries, particularly abdominoplasty, is evident as more patients seek treatment for the aftereffects of bariatric surgery. It is crucial to address the nutritional deficiencies often found in obese patients, as these can significantly impact wound healing and recovery post-surgery. Preoperative evaluations should also carefully consider potential complications such as cardiac and lung diseases, as well as deep venous thrombosis and clotting abnormalities. A key recommendation for patients is to cease smoking at least 3 months prior to surgery, as tobacco use can adversely affect the healing process. Furthermore, it is advisable for patients to have achieved their weight loss goals following bariatric surgery before undergoing abdominoplasty to ensure the best possible outcomes. Tracing the history of abdominoplasty reveals its century-long evolution. The procedure was first described by Kelly in 1899 [13]. Since then, numerous surgeons, including Thorek [14] and Pitanguy [15], have contributed to refining the technique, enhancing both its safety and aesthetic results. These historical advancements have shaped modern abdominoplasty into a more effective and safer procedure, benefiting a growing number of patients seeking post-bariatric body contouring [2,13,14,16,17].

Body contouring surgery, commonly pursued after significant weight loss or for cosmetic enhancements, offers a range of benefits from improved mobility and reduced infection rates to enhanced psychosocial wellbeing [6,9,15]. However, it is crucial to acknowledge the inherent risks involved. The literature has extensively discussed potential complications such as wound dehiscence, seroma, hematoma, and less than ideal aesthetic outcomes, underlining the importance of careful surgical planning and precise execution [9,15,18,19,20,21,22,23]. Research, including a notable study by Payam et al., has highlighted complication rates in post-bariatric body contouring, pinpointing specific risk factors like elevated BMI and smoking habits. In particular, abdominoplasty is noted for its relatively high complication rates, with figures from sources like the CosmetAssure database showing a range from 9.7% to 37.4%. This underscores the necessity for comprehensive risk assessment and detailed patient counseling before proceeding with such procedures [24,25,26,27,28,29].

Echoing these findings, Marouf and Mortada reported an overall complication rate of 38.2%, with specific complications including 1.0% partial necrosis, 1% scar asymmetries requiring surgical revision, 4.3% local infections, and 9.6% wound dehiscence. Additionally, they observed hematoma rates between 1.4% and 3.2%, seroma formation ranging from 13.3% to 16.4%, and instances of venous thromboembolism and pulmonary embolism at 0.3% each. Seroma was the most common complication at a rate of 15.4%, a figure that aligns with data from Marouf et al. and Marchica et al. [30,31], who reported an average rate of 13.1%. As highlighted in various studies, the frequency of wound dehiscence further emphasizes the complexities and challenges inherent in these surgical procedures [15,22,32,33].

Technological advancements have significantly transformed the realm of post-bariatric body contouring. The introduction of energy-based tools like ultrasound, electromagnetic devices, and radiofrequency instruments has markedly improved surgical precision and results [9,34,35]. However, these cutting-edge technologies are not without limitations, as issues of accessibility and affordability often preclude their widespread adoption among healthcare providers and patients. Notably, there is a clear link between higher preoperative BMI and reduced post-operative satisfaction, as observed by both patients and surgeons a year after the surgery. This correlation echoes other studies suggesting a strong connection between preoperative BMI and postoperative complications, potentially impacting satisfaction levels [36,37,38]. Higher BMI is frequently associated with increased surgical risks and prolonged recovery, leading to a gap between expected and actual outcomes. This underscores the need for tailored surgical protocols, particularly for patients who have undergone significant weight fluctuations following bariatric surgery. Future research should focus on devising evidence-based strategies to minimize the negative impact of high BMI on postoperative outcomes and improve overall patient satisfaction.

At our institutions, the management of postoperative complications in abdominoplasty, particularly seroma, follows a rigorously evidence-based approach [39]. Recent meta-analyses have critically assessed the effectiveness of various techniques purported to reduce seroma incidence, including the use of fibrin glue and quilting sutures [40]. Notably, a systematic review examining the impact of progressive tension sutures, preservation of Scarpa’s fascia, and the application of fibrin glue found that while some techniques notably reduced seroma formation, the application of fibrin glue did not demonstrate a significant benefit over standard abdominoplasty procedures (*p* > 0.05) [40]. Furthermore, a retrospective trial comparing the efficacy of the human fibrin sealant Artiss with progressive tension sutures using Stratafix showed that the use of fibrin sealant was associated with higher drain volumes and prolonged drain retention compared to PTS, indicating no advantage in seroma prevention or drain management. Given these findings, our institutional protocol continues to endorse the use of closed suction drains postoperatively, a method supported by extensive clinical application and outcome data [41]. Therefore, we have opted not to routinely incorporate quilting sutures or fibrin glue into our surgical practice due to the lack of compelling evidence from recent high-quality studies indicating significant advantages of these methods over traditional suction drainage [42]. This decision aligns with our commitment to providing care based on the highest standards of evidence, ensuring both safety and efficacy in patient outcomes.

This prospective study distinguishes itself in several respects. With a significant cohort of 500 patients, it provides a solid foundation for understanding post-bariatric abdominoplasty outcomes across varied clinical environments. The detailed collection of data, including BMI, weight loss, and specific comorbidities, facilitates a nuanced analysis of the factors affecting surgical results, aligning with insights from prior studies. Moreover, the methodical approach to post-operative evaluations, incorporating patient self-assessments and independent surgeon reviews, ensures a well-rounded perspective on the surgical outcomes. However, the study isn’t devoid of limitations. Its tertiary center nature, despite being a strength, may not fully represent the entire spectrum of clinical practices or patient demographics, somewhat narrowing its applicability to a wider context. The focus is primarily on quantitative measures, which might overlook the subjective or qualitative aspects of patients’ postoperative experiences. Finally, like many observational studies, it may not have entirely accounted for all confounding variables, which could influence the correlations noted in the findings.

## 5. Conclusions

The increasing prevalence of obesity highlights the growing need for bariatric surgeries and abdominoplasties to manage the aftermath of significant weight loss. Our study reveals a notable correlation between higher BMI and a heightened risk of postoperative complications, emphasizing the need for standardized protocols to effectively address the unique challenges faced by patients with elevated BMIs. Future research is essential to investigate the link between BMI and complication risks further and determine how uniform surgical guidelines can improve patient outcomes by reducing post-abdominoplasty complications.

## Figures and Tables

**Table 1:** Overview of patients’ characteristics.

Characteristics	Number (%)
Number of patients	500
Sex, female/male, no. (%)	382 (76.4)/118 (23.6)
Average age, median, range (years)	34.8, 25–61
Body mass index (BMI), median, range [kg/m^2^]	31.1, 25–48
Weight reduction, mean ± SD, range [kg]	60.23 ± 29.2, 30–89.5
The procedure of weight loss, no. of patients (%)	
Gastric banding	203 (40.6)
Gastric bypass	194 (38.8)
Gastric sleeve	80 (16.0)
Gastric bypass + sleeve	23 (4.6)
Smokers, no. (%)	110 (22%)
Type 2 diabetes, no. (%)	26 (5.2%)
Cardiovascular illnesses	73 (23.4)
Pulmonary illnesses	73 (14.6)

**Table 2:** Post-operative complication rates by procedure type.

Complications	Traditional Abdominoplasty n. 372 (74%)	Inverted-T Abdominoplasty N. 128 (26%)	Total	Mean BMI	*p*-Value	Diabetes
Wound infection	9 (1.8%)	11 (2.2%)	20	33.6	**0.005**	**3**
Wound dehiscence	20 (4%)	23 (4.6%)	43	34.7	**0.001**	**1**
Seroma	18 (3.6%)	24 (4.8%)	42	31.9	**0.005**	**0**
Hematoma	5 (1%)	8 (1.6%)	13	34.1	**0.005**	**0**
Tissue necrosis	0 (0%)	3 (0.6%)	3	31	0.44	4
Deep venous thrombosis	1 (0.2%)	0	1	29	0.5	3
Total	53 (10.6%)	69 (13.8%)				

**Table 3:** Multinomial correlation between BMI and complications. This table presents the regression coefficients showing the relationship between BMI and the rate of various complications. A higher BMI is associated with an increased rate of complications, as indicated by the bold values (with the exception of necrosis: coefficients < 0.03).

Complications	Coefficient
Seroma	
Age	0.829
BMI	**0.** **0001**
Weight reduction	0.073
Hematoma	
Age	0. 914
BMI	**0.** **002**
Weight reduction	0.077
Infection	
Age	0.124
BMI	**0.** **001**
Weight reduction	0.013
Dehiscence	
Age	0.147
BMI	**0.** **0001**
Weight reduction	0.320
Necrosis	
Age	0.789
BMI	0.445
Weight reduction	0.47

## Data Availability

The data presented in this study are available on request from the corresponding author.

## Supplementary Materials

The following supporting information can be downloaded at: https://www.mdpi.com/article/10.3390/jpm14070681/s1, Figure S1: Spearman’s rank Correlation. Figure S2. Logistic Regression of complication.

## Appendix A. Questionnaire

**The Questionnaire with a Four-Point Scale.**	**1: Poor**	**2: Fair**	**3: Good**	**4: Excellent**
1. I am satisfied with the size of the abdomen	○	○	○	○
2. I am satisfied with the shape of the abdomen	○	○	○	○
3. I am satisfied with the overall appearance of the abdomen	○	○	○	○
4. I am satisfied with the position of the umbilicus	○	○	○	○
**The questionnaire of surgeons.**	**Skin/fat excess (0/1-0/1)**		**Appearence of abdomen (0/1)**	**Umbilicus position (0/1)**
	○	○	○	○

## References

[1] Santos L.R.A., da Costa P.R., Maia T.S., Junior A.C., Resende V. (2023). Prospective cohort of parameters of glycemic and lipid metabolism after abdominoplasty in normal weight and formerly obese patiens. JPRAS Open.

[2] Cuomo R., Russo F., Sisti A., Nisi G., Grimaldi L., Brandi C., D’Aniello C. (2015). Abdominoplasty in Mildly Obese Patients (BMI 30–35 kg/m^2^): Metabolic, Biochemical and Complication Analysis at One Year. In Vivo.

[3] Aly A.S., Cram A.E., Heddens C. (2004). Truncal body contouring surgery in the massive weight loss patient. Clin. Plast. Surg..

[4] Brito I.M., Meireles R., Baltazar J., Brandao C., Sanches F., Freire-Santos M.J. (2020). Abdominoplasty and Patient Safety: The Impact of Body Mass Index and Bariatric Surgery on Complications Profile. Aesthetic Plast. Surg..

[5] Brower J.P., Rubin J.P. (2020). Abdominoplasty after Massive Weight Loss. Clin. Plast. Surg..

[6] Pierazzi D.M., Pica Alfieri E., Cuomo R., Bocchiotti M.A., Grimaldi L., Donniacuo A., Zerini I., Nisi G. (2022). Ligasure Impact and Ligasure Small Jaw in Body Contouring after Massive Weight Loss: A New Perspective. J. Investig. Surg..

[7] Pozzi M., Marcaccini G., Giardino F.R., El Araby M.M., Nisi G., Grimaldi L., Cuomo R. (2023). Flowchart in Post-Bariatric Surgery: A Research for the Appropriate Type and Timing of Plasties Reshaping the Body. Aesthetic Plast. Surg..

[8] Waldner F.A., Sadeghi P., Grimaldi L., Nisi G., Calomino N., Cuomo R. (2022). Tissue Adhesive: Current Uses and Strengths. J. Investig. Surg..

[9] Sadeghi P., Duarte-Bateman D., Ma W., Khalaf R., Fodor R., Pieretti G., Ciccarelli F., Harandi H., Cuomo R. (2022). Post-Bariatric Plastic Surgery: Abdominoplasty, the State of the Art in Body Contouring. J. Clin. Med..

[10] Nisi G., Giudice M., Bacchini S., Fasano G., Verre L., Cuomo R., Grimaldi L. (2022). To Keep or Not to Keep? The Hamletic Umbilical Dilemma: Preservation versus Reconstruction of the Umbilicus in Vertical Abdominoplasty. J. Clin. Med..

[11] Nisi G., Giardino F.R., Giudice M., Fasano G., Cuomo R., Grimaldi L. (2022). The Jaws Brachioplasty: An Original Technique: Improving Aesthetic Outcomes in Arm Lift Procedures. J. Clin. Med..

[12] Cuomo R., Giardino F.R., Nisi G., Brandi C., Zerini I., Voglino C., Gaggelli I., Grimaldi L. (2019). Aspiration Pneumonia: A Shadow in Post-Bariatric Patient: Correlation between aspiration and minigrastric bypass. Obes. Surg..

[13] Sisti A., Cuomo R., Zerini I., Tassinari J., Brandi C., Grimaldi L., D’Aniello C., Nisi G. (2015). Complications Associated with Medial Thigh Lift: A Comprehensive Literature Review. J. Cutan. Aesthet. Surg..

[14] Song A.Y., Jean R.D., Hurwitz D.J., Fernstrom M.H., Scott J.A., Rubin J.P. (2005). A classification of contour deformities after bariatric weight loss: The Pittsburgh Rating Scale. Plast. Reconstr. Surg..

[15] Losco L., Roxo A.C., Roxo C.W., de Sire A., Bolletta A., Cuomo R., Grimaldi L., Cigna E., Del Pino Roxo C. (2022). Helix Thigh Lift. A Novel Approach to Severe Deformities in Massive Weight Loss Patients. J. Investig. Surg..

[16] Sisti A., Cuomo R., Brandi C., Grimaldi L., D’Aniello C., Nisi G. (2017). Management of the Postbariatric Medial Thigh Deformity. Plast. Reconstr. Surg..

[17] Sisti A., Cuomo R., Milonia L., Tassinari J., Castagna A., Brandi C., Grimaldi L., D’aniello C., Nisi G. (2018). Complications associated with brachioplasty: A literature review. Acta Biomed..

[18] Cuomo R., Nisi G., Grimaldi L., Brandi C., Sisti A., D’Aniello C. (2015). Immunosuppression and Abdominal Wall Defects: Use of Autologous Dermis. In Vivo.

[19] Elfanagely O., Othman S., Mellia J.A., Messa C.A., Fischer J.P. (2021). Quality of Life and Complications in the Morbidly Obese Patient following Post-Bariatric Body Contouring. Aesthetic Plast. Surg..

[20] Garcia Botero A., Garcia Wenninger M., Fernandez Loaiza D. (2017). Complications After Body Contouring Surgery in Postbariatric Patients. Ann. Plast. Surg..

[21] Wiedner M., Richter D.F. (2021). Invited Discussion on: Quality of Life and Complications in the Morbidly Obese Patient following Post-Bariatric Body Contouring. Aesthetic Plast. Surg..

[22] Han J., Cuomo R., Zhao Y., Pan B., Yang Q. (2021). The Morphology and Bending Behavior of Regenerated Costal Cartilage with Kawanabe-Nagata Method in Rabbits—The Short Term Result of an Experimental Study. J. Investig. Surg..

[23] Khoshnevis J., Cuomo R., Karami F., Dashti T., Motamedi A.K., Motamedi M.K., Azargashb E., Aryan N., Sadeghi P. (2022). Jump Technique versus Seton Method for Anal Fistula Repair: A Randomized Controlled Trial. J. Investig. Surg..

[24] Grieco M., Grignaffini E., Simonacci F., Raposio E. (2015). Analysis of Complications in Postbariatric Abdominoplasty: Our Experience. Plast. Surg. Int..

[25] McCarty J.C., Lorenzi-Mendez R., Fruge S., Hamaguchi R., Colwell A.S. (2023). Does Concomitant Umbilical Hernia Repair Increase the Risk of Complications in Abdominoplasty? A Propensity Score Matched Analysis. Aesthet. Surg. J..

[26] Neaman K.C., Hansen J.E. (2007). Analysis of complications from abdominoplasty: A review of 206 cases at a university hospital. Ann. Plast. Surg..

[27] Rangaswamy M. (2013). Minimising complications in abdominoplasty: An approach based on the root cause analysis and focused preventive steps. Indian. J. Plast. Surg..

[28] Rosenfield L.K. (2021). Commentary on: Post-Bariatric Abdominoplasty: Analysis of 406 Cases with Focus on Risk Factors and Complications. Aesthet. Surg. J..

[29] Schlosshauer T., Kiehlmann M., Jung D., Sader R., Rieger U.M. (2021). Post-Bariatric Abdominoplasty: Analysis of 406 Cases with Focus on Risk Factors and Complications. Aesthet. Surg. J..

[30] Marchica P., Costa A.L., Brambullo T., Marini M., Masciopinto G., Gardener C., Grigatti M., Bassetto F., Vindigni V. (2023). Retrospective Analysis of Predictive Factors for Complications in Abdominoplasty in Massive Weight Loss Patients. Aesthetic Plast. Surg..

[31] Marouf A., Mortada H. (2021). Complications of Body Contouring Surgery in Postbariatric Patients: A Systematic Review and Meta-Analysis. Aesthetic Plast. Surg..

[32] Casella D., Fusario D., Cassetti D., Pesce A.L., De Luca A., Guerra M., Cuomo R., Ribuffo D., Neri A., Marcasciano M. (2022). Controlateral Symmetrisation in SRM for Breast Cancer: Now or Then? Immediate versus Delayed Symmetrisation in a Two-Stage Breast Reconstruction. Curr. Oncol..

[33] Casella D., Palumbo P., Sandroni S., Caponi C., Littori F., Capuano F., Grimaldi L., Marcasciano M., Cuomo R. (2021). Positive ROS (Reactive Oxygen Species) Modulator Engineered Device Support Skin Treatment in Locally Advanced Breast Cancer (LABC) Enhancing Patient Quality of Life. J. Clin. Med..

[34] Cuomo R., Pieretti G., Ciccarelli F. (2023). Letter on “An Original Approach to Massive Weight Loss Deformities in the Lower Thigh: A Retrospective Assessment of Results and Patients”. Aesthetic Plast. Surg..

[35] Voglino C., Tirone A., Ciuoli C., Benenati N., Bufano A., Croce F., Gaggelli I., Vuolo M.L., Badalucco S., Berardi G. (2021). Controlling Nutritional Status (CONUT) Score and Micronutrient Deficiency in Bariatric Patients: Midterm Outcomes of Roux-en-Y Gastric Bypass Versus One Anastomosis Gastric Bypass/Mini Gastric Bypass. Obes. Surg..

[36] Sisti A., Huayllani M.T., Boczar D., Restrepo D.J., Cinotto G., Lu X., Cuomo R., Grimaldi L., Nisi G., Forte A.J. (2021). Umbilical Reconstruction Techniques: A Literature Review. Aesthetic Plast. Surg..

[37] Cuomo R., Grimaldi L., Nisi G., Zerini I., Giardino F.R., Brandi C. (2021). Ultraportable Devices for Negative Pressure Wound Therapy: First Comparative Analysis. J. Investig. Surg..

[38] Voglino C., Tirone A., Ciuoli C., Benenati N., Paolini B., Croce F., Gaggelli I., Vuolo M.L., Cuomo R., Grimaldi L. (2020). Cardiovascular Benefits and Lipid Profile Changes 5 Years After Bariatric Surgery: A Comparative Study Between Sleeve Gastrectomy and Roux-en-Y Gastric Bypass. J. Gastrointest. Surg..

[39] Ardehali B., Fiorentino F. (2017). A Meta-Analysis of the Effects of Abdominoplasty Modifications on the Incidence of Postoperative Seroma. Aesthetic Surg. J..

[40] Zeplin P.H., Langer S., Schwarzenberger S., Spindler N. (2021). Fibrin Sealant Artiss Compared to Progressive Tension Sutures with Stratafix in the Management of Wound Drainage Following Post-Bariatric Body-Contouring Surgery. Plastic Surg..

[41] Ciccarelli F., Pieretti G., Seth I. (2024). The Effect of Drains and Compressive Garments Versus Progressive Tensioning Sutures on Seroma Formation in Abdominoplasty: A New Perspective for Abdominoplasty Procedure?. Aesthetic Plastic Surg..

[42] Chua M., Seth I., Tobin V., Kaplan E., Rozen W.M. (2024). The Preservation of Umbilical Blood Supply in Combined Ventral Hernia Repair and Abdominoplasty: A Narrative Review. Aesthetic Plastic Surg..

