# Supplementary material for: Experience in Post-Bariatric Abdominoplasty for Patients with Significant Weight Loss: A Prospective Study
Source: J Pers Med. 2024 Jun 25;14(7):681. doi: 10.3390/jpm14070681 (PMC11278261; doi:10.3390/jpm14070681)
Supplement: Supplementary file 1 [file jpm-14-00681-s001.zip › jpm-3041327-supplementary.pdf]

(obs=500)

Key
rho<br>*Sig. level*

	Et	BMI	Riduzi~g	Scorepaz	Scorech1	Scorech2
Et	1.0000					
BMI	0.1186<br>0.0080	1.0000				
Riduzionep~g	-0.0058<br>0.8978	0.0066<br>0.8838	1.0000			
Scorepaz	0.0159<br>0.7221	-0.0864<br>0.0536	-0.0136<br>0.7612	1.0000		
Scorech1	0.0446<br>0.3194	-0.0232<br>0.6043	0.0058<br>0.8966	0.0570<br>0.2032	1.0000	
Scorech2	0.0251<br>0.5751	0.0346<br>0.4400	-0.0298<br>0.5056	-0.0050<br>0.9110	0.0044<br>0.9211	1.0000

Figure S1. Spearman's rank Correlation

Measure	Value
Number of observations	500
LR chi2(7)	301.26
Prob > chi2	<0.0001
Log likelihood	-128.32086
Pseudo R^2	0.5400

Regression Coefficients

Predictor	Coefficient	Std. Err.	z-score	P>|z|	95% Conf. Interval
Age	-0.0095996	0.0209327	-0.46	0.647	[-0.0506269, 0.0314
BMI	1.700768	0.1789433	9.50	0.000	[1.350046, 2.05149]
Scorepat	-0.3765009	0.3052322	-1.23	0.217	[-0.9747451, 0.2217
Scoresur1	-0.0901613	0.3170958	-0.28	0.776	[-0.7116576, 0.5313
Scoresur2	0.0715775	0.3227459	0.22	0.824	[-0.5609927, 0.7041
Comorbidities	0.5537552	0.1378376	4.02	0.000	[0.2835985, 0.82391
Weight reduction	-0.0139285	0.0091543	-1.52	0.128	[-0.0318706, 0.0040
Constant (_cons)	-52.96865	5.866654	-9.03	0.000	[-64.46708, -41.470

Figure S2. Logistic Regression of complication
